# Retrospective analysis of Acinetobacter baumannii bacteraemia risk factors, complications and mortality in a tertiary university hospital in Saudi Arabia

**DOI:** 10.1099/acmi.0.000826.v4

**Published:** 2024-09-30

**Authors:** Reham Kaki

**Affiliations:** 1 Department of Medicine, King Abdulaziz University, Jeddah, Saudi Arabia; 2 Department of Infectious Disease & Infection Control and Environmental Health, King Abdulaziz University Hospital, Jeddah, Saudi Arabia

**Keywords:** *Acinetobacter*, antibiotics, bacteraemia, mortality, resistance, risk factors

## Abstract

**Introduction.** There are many multidrug-resistant isolates of the nosocomial pathogen, *Acinetobacter baumannii*, causing severe healthcare-acquired infections in terminally ill patients with high mortality and morbidity rates.

**Aim.** This study aims to retrospectively analyse *A. baumannii* bacteraemia (ABB) cases in Saudi Arabia, where the information is sparse regarding the prevalence, risk factors, clinical disease, antibiotic regimen, antibiotic susceptibility, treatment outcomes and mortality associated with this infection.

**Methods.** A retrospective chart review was conducted between 1 January 2015 and 31 December 2022 to identify all patients aged 14 years and above with ABB. Demographic and clinical data, as well as results from laboratory analyses, were collected from patients’ electronic charts. Statistical analyses were performed on the data to identify factors associated with 90-day mortality.

**Results.** Of the 122 ABB cases, 71 (63.4%) died. The factors that were found to be associated with 90-day mortality were the Charlson Comorbidity Index, Pitt bacteraemia score, quick Sequential Organ Failure Assessment score (*P*<0.001 for each), hospital ward (*P*<0.02), short duration of antibiotic treatment (*P*<0.01) and higher age (*P*<0.05). The most common source of infection was central line-associated bloodstream infection in 52.7%. Also associated with mortality were inappropriate antimicrobial therapy (*P*<0.02) and empirical use of colistin (*P*<0.05). In many patients, ABB was caused by carbapenem-resistant *A. baumannii* [(CRAB), 69.6%], and 74.4% of those patients died.

**Conclusion.** To prevent ABB-associated mortality, an appropriate regimen and duration of treatment are necessary. Hospitals should also practice proper hygiene to prevent the spread of ABB. CRAB is a growing threat in hospitals in Saudi Arabia, especially in the critical care setting, and carries a very high risk of mortality.

## Data summary

No supporting external data were generated for this work. The author has provided all supporting data within the article.

## Introduction

*Acinetobacter* is a Gram-negative, strictly aerobic, catalase-positive and oxidase-negative coccobacillus that consists of over 50 species that are mostly non-pathogenic environmental organisms. However, a few species of *Acinetobacter,* such as *Acinetobacter baumannii*, have emerged as a threat to human health, potentially causing severe infections primarily in hospital settings [1,2]. A vast majority of these infections in humans are caused by the virulent *A. baumannii* [1,3] although infections with other *Acinetobacter* species, such as *Acinetobacter nosocomialis*, *Acinetobacter lwoffii*, *Acinetobacter pittii* and *Acinetobacter radioresistens*, have also been reported [3,6].

*A. baumannii* is a major contributing factor to many healthcare-acquired infections (HAIs) in terminally ill patients [7]. Over the years, *A. baumannii* has acquired antimicrobial resistance on a large scale, leading to a huge burden on hospital costs and threatening patient safety [8,10]. In one study, more than 18% of *A. baumannii* strains were found to be resistant to first-line antibiotics, such as β-lactams, fluoroquinolones and carbapenems [11]. *A. baumannii* confers elevated mortality risk during the first 30–90 days following infection, in comparison to other Gram-negative and Gram-positive multidrug-resistant (MDR) bacteria [12]. Bloodstream infections with *A. baumannii* are linked with a mortality rate of up to 59% [13] and have the third highest crude mortality rate in the intensive care unit (ICU), succeeded only by *Pseudomonas aeruginosa* and *Candida* spp. infections [14]. Moreover, the mortality rate in children with carbapenem-resistant *A. baumannii* (CRAB) infection is 30.9% [15], emphasizing the need for the discovery of new agents to which *A. baumannii* is susceptible. According to the United States Centers for Disease Control (2019), *A. baumannii* remains an imminent threat due to the extensively resistant strains, which have been isolated and are associated with high mortality rates [16]. In the last few years, a pan-drug-resistant strain has also emerged [17,18].

Several factors are implicated in *A. baumannii* transmission and the emergence of its resistance. These include prolonged hospitalization, increased usage of wide-spectrum antimicrobials (especially third-generation cephalosporins, carbapenems and fluoroquinolones), long-term stay in a healthcare facility and lapses in cleaning and disinfection [19,24]. In hospitals, particularly in ICUs, inappropriate disinfection of mobile medical equipment and poor hand hygiene contribute to the transmission of *A. baumannii*, which has been responsible for several outbreaks [25,29]. This organism is capable of surviving on dry surfaces for a period of up to 5 months, which is an obstacle for infection control strategies in hospitals [30]. The most frequent nosocomial *A. baumannii* infections are ventilator-associated pneumonia, followed by bloodstream infections, meningitis, neurosurgical procedures, infections of soft tissue and skin resulting from burns and infections of the intra-abdominal region and urinary tract [6,31,35].

Information regarding the prevalence, risk factors, clinical disease, treatment outcomes and mortality associated with *A. baumannii* bacteraemia (ABB) in Saudi Arabia is sparse. Therefore, the central purpose of the study was to evaluate ABB cases and explore the risk factors that led to such infection, the clinical diseases they had, and their treatment response.

## Methods

### Patients and settings

This study was conducted at King Abdulaziz University Hospital, a tertiary hospital consisting of 1000 beds. A retrospective chart review was conducted between 1 January 2015 and 31 December 2022 to select all patients aged 14 years or above with *Acinetobacter*-positive blood cultures. This was done by accessing medical records for all blood cultures and filtering for positive results. Patients below 14 years of age were excluded.

### Ethical approval

The ethical approval for the use of the patient information for this study was granted by the ethical review committee (Unit of Biomedical Ethics, Research Ethics Committee) of King Abdulaziz University (reference number 301-23). The study methodology was retrospective, and hence, the requirement for obtaining informed consent was waived. To assure confidentiality, all obtained medical records were stored on a password-secured personal computer. Only the information needed for the study was attained, and each individual was assigned a number to identify them.

### Data collection

We collected information from patient electronic charts that included date of birth, gender, body mass index (BMI), duration of hospital stay, clinical symptoms, admissions in the ICU, culture results, comorbidities and chronic illnesses, human immunodeficiency virus status, Severe Acute Respiratory Syndrome Coronavirus 2(SARS-CoV-2) infection status, surgical history, type and duration of antimicrobial used, mortality within 90 days of the bacteraemia diagnosis and the results of laboratory work. Laboratory work included haemoglobin level, white cell count, platelet count, electrolytes and coagulation profile. Infections that occurred 48 h or more after hospitalization were defined as HAI. Infections that developed in less than 48 h of admission to hospitals were defined as community-acquired infections.

Phenotypic identification of all *Acinetobacter* blood isolates was conducted by Gram staining followed by biochemical assays: catalase and oxidase tests. For susceptibility testing, the VITEK 2 (bioMérieux, France) automated system was used as per the manufacturer’s instructions. A microbial suspension, calibrated to the 0.5 McFarland standard, was applied to a susceptibility testing card (Antimicrobial Susceptibility Testing-N376). Antibiotics that were tested included meropenem, ceftriaxone, cefepime, amikacin, ciprofloxacin, gentamicin, imipenem, ceftazidime, tigecycline, piperacillin–tazobactam, cefotaxime, trimethoprim–sulfamethoxazole and colistin. The minimum inhibitory concentration for colistin was defined at >2 mg l^−1^ . Confirmation of colistin resistance was done by performing an E-test, and validation was carried out using the broth microdilution technique. All procedures were performed in accordance with the guidelines of the Clinical and Laboratory Standards Institute [36,39]. CRAB was defined as an isolate resistant to a carbapenem antimicrobial, e.g. meropenem or imipenem.

Treatment consisting of a minimum of one antibiotic to which the *Acinetobacter* isolate was susceptible was defined as the appropriateness of treatment. As some of the patients died before susceptibility testing could be performed, so its susceptibility testing was not performed. In those patients, the appropriateness of treatment was considered to be unknown.

### Statistical analysis

Data were checked for errors and missing data. Descriptive statistics were performed to present all studied categorical and numerical variables in terms of their percentage frequency. Categorical variables were analysed using Pearson’s chi-square test to assess associations between different variables. Fisher’s exact test was employed to identify the level of significance amongst categorical variables with small sample sizes. As for the analysis of numerical variables, two-sided *t*-tests were performed. A *P*-value of ≤ 0.05 was considered statistically significant. All statistical analyses were conducted using Statistiscal Package of Social Science (SPSS) version 24.0 at a confidence interval of 95%, which is the standard practice. In each stage of calculation, a 95% confidence interval was specified on the SPSS dialogue box.

## Results

### Patient demographics

A total of 112 ABB cases were detected in this retrospective chart review. Age ranged from 15 to 89 years, with a mean of 58 years (sd, 17.6). The study population comprised 74 (66.1%) males and 38 (33.9%) females. The mean BMI ranged from 17 to 47 kg m^−2^, with a mean of 27 kg m^−2^ (sd, 5.9). Most of the patients were from the medical ICU [MICU (54.5%)], followed by the emergency department (16.1%), medical ward (15.2%), surgical ward (8.0%), surgical ICU (3.6%) and dialysis unit (2.7%). Of all the ABB cases, 41 (36.6%) patients survived, while 71 (63.4%) died.

### Factors associated with mortality

The association of mortality with categorical variables such as sex, location in the hospital and BMI were analysed. Of these variables, only location was associated with mortality (*P*<0.02), with the highest number of deaths (*n* = 44) observed among MICU-admitted cases (Table 1). It can be because the different hospital units tend to different types of illnesses that vary in severity. Therefore, mortality may be higher in units where patients with critical illnesses are generally found. It is also the reason why MICU-admitted patients have the highest mortality due to the critical nature of conditions treated there.

**Table 1.** Distribution of cases by sex, location and BMI category and its association with mortality

		Total, *n* (%)	Mortality		
**Variables**	**Attributes**		**No, *n* (%**)	**Yes, *n* (%**)	*P*-value*
**Sex**	Male	74 (66.1)	27 (36.5)	47 (63.5)	0.970
	Female	38 (33.9)	14 (36.8)	24 (63.2)	
**Location**	MICU	61 (54.5)	17 (27.9)	44 (72.1)	<0.02
	ED	18 (16.1)	9 (50.0)	9 (50.0)	
	Medical ward	17 (15.2)	6 (35.3)	11 (64.7)	
	Surgical ward	9 (8.0)	6 (66.7)	3 (33.3)	
	SICU	4 (3.6)	0 (0.0)	4 (100.0)	
	Dialysis	3 (2.7)	3 (100.0)	0 (100.0)	
**BMI category†**	Underweight	1 (0.9)	1 (100.0)	0 (0.0)	0.788
	Healthy	37 (33.0)	15 (40.5)	22 (59.5)	
	Overweight	42 (37.5)	15 (35.7)	27 (64.3)	
	Obesity class 1	19 (17.0)	6 (31.6)	13 (68.4)	
	Obesity class 2	7 (6.3)	2 (28.6)	5 (71.4)	
	Obesity class 3	6 (5.4)	2 (33.3)	4 (66.7)	

* Pearson’s chi-square test.

† BMI categories: A healthy BMI was defined as a BMI from ≥18.5 to <25 kg m−2, and overweight was defined as a BMI from ≥25 to <30 kg m−2. Obesity categories were defined as class 1: BMI from ≥30 to <35 kg m−2, class 2: BMI from ≥35 to <40 kg m−2 and class 3: BMI ≥40 kg m−2.

BMIbody mass indexEDemergency departmentMICUmedical intensive care unit*n*number of patientsSICUsurgical intensive care unit

Association with mortality was also analysed for all numerical variables. Three types of assessments were performed for underlying diseases and disease severity: the Charlson Comorbidity Index, Pitt bacteraemia score and quick Sequential Organ Failure Assessment (qSOFA) score. The mean and standard deviations for the three scores are presented in Table 2. The survivors had lower scores for these three assessments compared to their counterparts, with all *P*-values < 0.001. There was a statistically significant association between advanced age and mortality (*P*=<0.05). Additionally, a shorter duration of antibiotic therapy was linked to a higher mortality (*P*=<0.01) (Table 2).

**Table 2.** All studied numerical variables and their relationship with mortality

Variables	*n*	All cases		Alive		Dead		*P*-value†
		Mean	sd	Mean	sd	Mean	sd	
Age, in years	112	57.97	17.62	52.98	19.26	60.86	16.04	<0.05
BMI, in kg m^−2^	111	27.12	5.90	26.17	5.59	27.67	6.05	0.189
LOS, in days	99	31.13	21.75	32.73	22.70	30.43	21.46	0.640
Charlson Comorbidity Index	112	3.54	2.36	2.49	1.87	4.15	2.40	<0.001
Pitt bacteraemia score	112	5.52	2.67	3.61	2.47	6.62	2.11	<0.001
qSOFA score	112	2.37	1.05	1.56	1.27	2.83	0.48	<0.001
Duration of antibiotic treatment, in days	112	14.29	15.12	19.85	18.09	11.07	12.11	<0.01

* BMI was not known for one case.

† Two-sided *t*-test.

BMIbody mass indexLOSlength of hospital stay*n*number of patientsqSOFAquick sequential organ failure assessment

Of the 71 patients who died within 90 days after ABB diagnosis, 39.4% (*n*=28/71) died within 3 days after diagnosis, 53.5% (*n*=38/71) between 4 and 30 days after diagnosis and 7.0% (*n*=5/71) between 31 and 90 days after diagnosis.

### Clinical signs and symptoms and laboratory analyses of ABB patients

The most common clinical features among the patients were shock (69.6%) and fever (42.9%). Of the features based on the laboratory analyses (Table 3), anaemia (95.5%) was the most common followed by hypoalbuminaemia (69.6%), elevated d-dimer (57.1%), prolonged International Normalized Ratio (INR) (54.5%), leucocytosis (51.8%), hyperglycaemia (47.3%) and thrombocytopaenia (44.6%). Other symptoms were found in only a small number of patients. Significant associations were found between mortality and thrombocytopaenia (*P*<0.001), hypoalbuminaemia (*P*<0.01), prolonged INR (*P*<0.05) and hyperkalaemia (*P*<0.05). The other features were not significantly related to mortality.

**Table 3.** Relationship between laboratory values and mortality

Sources	All cases	Mortality		
	*N* = **112**	No, *N* = **41**	Yes, *N* = **71**	*P*-value*
	*n* (%)	*n* (%)	*n* (%)	
Anaemia	107 (95.5)	38 (92.7)	69 (97.2)	0.267
Leucopenia	19 (17.0)	6 (14.6)	13 (18.3)	0.618
Leucocytosis	58 (51.8)	21 (51.2)	37 (52.1)	0.927
Thrombocytopaenia	50 (44.6)	9 (22.0)	41 (57.7)	<0.001
Hypoglycaemia	9 (8.0)†	0 (0.0)	9 (12.7)	na
Hyperglycaemia	53 (47.3)‡	15 (34.1)	38 (53.5)	na
Hyperkalaemia	12 (10.7)	1 (2.4)	11 (15.5)	0.031
Hyponatraemia	31 (27.7)	12 (29.3)	19 (26.8)	0.775
Hypoalbuminaemia	78 (69.6)	22 (53.7)	56 (78.9)	0.008
Prolonged INR	61 (54.5)§	16 (3.9)	45 (63.4)	0.014
Elevated d-dimer	64 (57.1)¶	16 (39.0)	48 (67.6)	na
Lactic acid	39 (34.8)**	4 (9.8)	35 (49.3)	na

na,. not determined due to the number of missing samples.

*N*: Represents the total number of cases in each category. For example, "*N* = 112" indicates that there are a total of 112 cases in the study.

*n*: Refers to the number of cases with a specific feature or condition within the total. For example, "*n* (%)" shows the count and percentage of cases with that feature among the total cases or within the specified mortality category (No or Yes).

* Pearson’s chi-square test.

† Fifty-seven samples were not analysed.

‡ Thirty samples were not analysed.

§ Five samples were not analysed.

¶ Thirty-five samples were not analysed.

** Thirty-nine samples were not analysed.

The frequency distributions of potential risk factors, such as comorbidities, are presented in Table 4. The most common comorbidity was renal impairment in 66.1%. Several potential risk factors were found to be associated with mortality: renal impairment, ICU stay, use of wide-spectrum antibiotics, anti-methicillin-resistant *Staphylococcus aureus* treatment and the use of mechanical ventilation (all at *P*<0.001), haemodialysis (*P*<0.02), presence of an indwelling device (*P*<0.05) and the empiric use of colistin (*P*<0.05) (Table 4). These treatments and comorbidities represent serious underlying conditions, which themselves are associated with higher mortality. Nonetheless, together with *A. baumannii*, these factors can significantly worsen patient conditions and outcomes.

**Table 4.** Comorbidities and other potential risk factors in the patients

		Total	Mortality		
Variables	Attributes	*n* (%)	No, *n* (%)	Yes, *n* (%)	*P*-value*
Diabetes mellitus	No	58 (51.8)	23	35	0.488
	Yes	54 (48.2)	18	36	
Hypertension	No	57 (50.9)	22	35	0.656
	Yes	55 (49.1)	19	36	
Renal impairment	No	38 (33.9)	24	14	<0.001
	Yes	74 (66.1)	17	57	
Haemodialysis	No	57 (50.9)	27	30	<0.02
	Yes	55 (49.1)	14	41	
Heart disease	No	65 (58.0)	27	38	0.203
	Yes	47 (42.0)	14	33	
CLD	No	109 (97.3)	41	68	0.298^†^
	Yes	3 (2.7)	0	3	
CVS disease	No	88 (78.6)	30	58	0.290
	Yes	24 (21.4)	11	13	
Malignancy	No	95 (84.8)	38	57	0.103†
	Yes	17 (15.2)	3	14	
	Haematological malignancies				
	- Large B-cell lymphoma	3			
	- Hodgkin’s lymphoma	2			
	- Multiple myeloma	2			
	- Myelodysplastic syndrome	1			
	- AML	1			
	- Follicular non-Hodgkin’s lymphoma	1			
	Solid organ malignancies				
	- Endometrial cancer	2			
	- Ovarian cancer	2			
	- Mandible cancer	1			
	- Neuroendocrine tumour	1			
	- Rectal cancer	1			
ICU stay	No	26 (23.2)	18	8	<0.001
	Yes	86 (76.8)	23	63	
Broad-spectrum antibiotic	No	18 (16.1)	13	5	<0.001
	Yes	93 (83.0)	27	66	
	Not mentioned	1 (0.9)	1	0	
Anti-MRSA treatment	No	22 (19.6)	16	6	<0.001
	Yes	89 (79.5)	24	65	
	Not mentioned	1 (0.9)	1	0	
Mechanical ventilation	No	27 (24.1)	18	9	<0.001
	Yes	85 (75.9)	23	62	
Indwelling device	No	8 (7.1)	6	2	<0.05†
	Yes	104 (92.9)	35	69	
Surgical history	No	85 (75.9)	29	56	0.332
	Yes	27 (24.1)	12	15	
Immunosuppression	No	42 (37.5)	18	24	0.977
	Yes				
	- Steroids	58 (51.8)			
	- Steroids and tocilizumab	6 (5.4)			
	- Steroids and chemotherapy	3 (2.7)			
	- Chemotherapy	2 (1.8)			
	Not mentioned	1 (0.9)	1	0	
HIV	No	111 (99.1)	40	71	0.366†
	Not tested	1 (0.9)	1	0	
Empiric colistin	No	37 (33.0)	19	18	<0.05
	Yes	75 (67.0)	22	53	

* Pearson’s chi-square test except where otherwise indicated.

† Fisher’s exact test.

AMLacute myeloid leukaemiaCLDchronic liver diseaseCVScardiovascular diseaseHIVhuman immunodeficiency virusICUintensive care unitMRSAmethicillin-resistant *Staphylococcus aureus*

Since the study period also included the time of the COVID-19 pandemic, diagnostic tests to detect SARS-CoV-2 infection were performed in 68 of the ABB cases. Among the patients tested, 30 (44.1%) were found to be infected with SARS-CoV-2, whereas in 38 (55.9%), no infection was detected. Out of 30 SARS-CoV-2 patients, 24 died, and out of 38 SARS-CoV-2 -negative patients, 24 died. There was no association with increased mortality (*P* = 0.052).

### Pathogen cultures

In addition to *A. baumannii*-positive blood cultures, for many patients, *A. baumannii* was also cultured from other tissues. Respiratory fluid [sputum (44.6%)] was found to be the primary culture site, followed by wound tissue in five (4.5%) cases, both respiratory and wound tissue in five (4.5%) cases, ascitic fluid in two (1.8%) cases and cerebrospinal fluid in one case (0.9%). In 49 cases (43.8%), *A. baumannii* was not cultured from other tissues. A statistically insignificant association was found between the culture site and mortality (*P*=0.442).

The patients were infected by three types of *Acinetobacter* — *A. baumannii* (in 27), *A. lwoffii* (in 7) and CRAB (in 78). In total, 63.4% of the patients died (*n* = 71/112). None of the *A. lwoffii* cases died (0%, *n*=0/7), and 48.1% of the *A. baumannii* cases died (*n* = 13/27). In contrast, 74.4% (*n*=58/78) of the CRAB cases died, which was significantly more than the other two types (*P*<0.001).

The sources of ABB are presented in Table 5. It shows that the predominant source of ABB was central line-associated bloodstream infection (CLABSI) in 52.7% of the patients. The highest mortality was observed among the patients with healthcare-acquired pneumonia (70.4%). Mortality data were not significantly different for the source of infection *(P*=0.517) (Table 5).

**Table 5.** Relationship between types of infection and mortality

Sources	All cases	Mortality		
	*N* = **112**	No, *N* = **41**	Yes, *N* = **71**	*P*-value**
	*n* (%)	*n* (%)	*n* (%)	
CLABSI	*59*(52.7)	21 (35.6)	38 (64.4)	0.517
Healthcare-acquired pneumonia	27 (24.1)	8 (29.6)	19 (70.4)	
Skin and soft-tissue infections	13 (11.6)	5 (32.3)	8 (61.5)	
Other sources	13 (11.6)	7 (53.8)	6 (46.2)	
- Primary bacteraemia	5	5	0	
- Abdominal infection	2	1	1	
- External ventricular drain infection	1	1	0	
- Unknown	5	0	5	

"*n*" represents the number of cases with a specific infection source among the total cases.

* Pearson’s chi-square test was performed to determine the significance.

CLABSIcentral line-associated bloodstream infection*N*number of patients

**Fig. 1.**
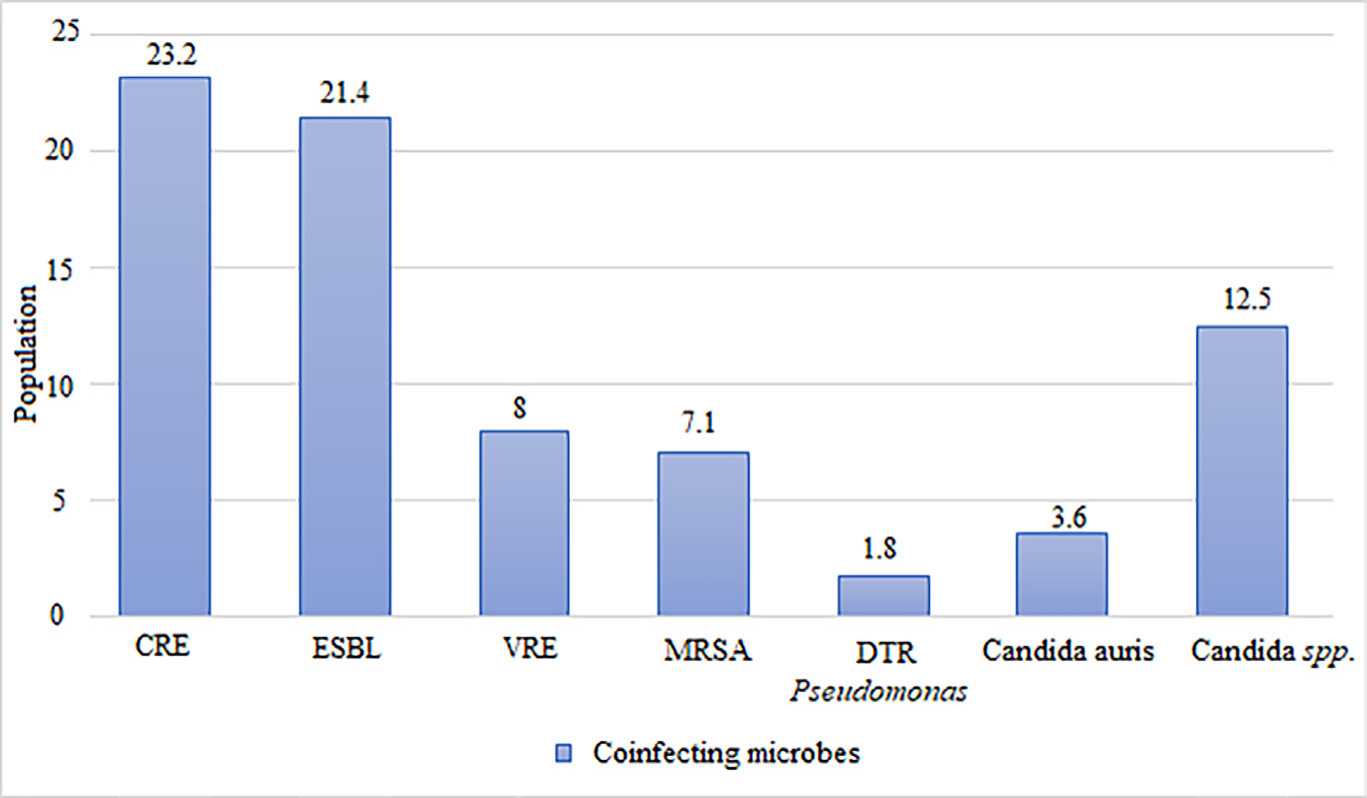
Number and percentage of other pathogens present. The ESBL bacteria were a mix of *Escherichia coli* and *Klebsiella*. **Abbreviations:** CRE, carbapenem-resistant Enterobacteriaceae; DTR, difficult-to-treat resistance; ESBL, extended-spectrum β-lactamase; MRSA, methicillin-resistant *Staphylococcus aureus*; VRE, vancomycin-resistant *Enterococci*.

Many of the patients were also co-infected with other pathogens. The most common co-infecting microbe was carbapenem-resistant Enterobacteriaceae (CRE) in 23.2% of patients, followed by Gram-negative organisms producing extended-spectrum β-lactamase (ESBL) in 21.4% of patients (Fig. 1).

### Antibiotic susceptibility and treatment

An investigation of the susceptibility of isolates against various relevant antibiotics was also carried out. Many isolates were found to be resistant to several antibiotics, such as the combination of piperacillin and tazobactam and the combination of trimethoprim, sulfamethoxazole, meropenem, ciprofloxacin, cefepime, gentamicin, imipenem and ceftazidime (Table 6).

**Table 6.** Antibiotics culture and its susceptibility

Antibiotic name	C/S report	*n*	%
Meropenem	Resistant	77	68.8
	Susceptible	26	23.2
	Not tested	9	8.0
Piperacillin and tazobactam	Resistant	82	73.2
	Susceptible	20	17.9
	Not tested	10	8.9
Ciprofloxacin	Resistant	79	70.5
	Susceptible	24	21.4
	Not tested	9	8.0
Cefepime	Resistant	77	68.8
	Intermediate	1	0.9
	Susceptible	21	18.8
	Not tested	13	11.6
Gentamicin	Resistant	57	50.9
	Intermediate	2	1.8
	Susceptible	42	37.5
	Not tested	11	9.8
Imipenem	Resistant	76	67.9
	Intermediate	1	0.9
	Susceptible	12	10.7
	Not tested	23	20.5
Trimethoprim and sulfamethoxazole	Resistant	74	66.1
	Susceptible	26	23.2
	Not tested	12	10.7
Ceftazidime	Resistant	78	69.6
	Intermediate	1	0.9
	Susceptible	23	20.5
	Not tested	10	8.9
Colistin	Resistant	5	4.5
	Susceptible	31	27.7
	Not tested	76	67.9
Tigecycline	Resistant	2	1.8
	Intermediate	2	1.8
	Susceptible	9	8.0
	Not tested	99	88.4
Amikacin	Resistant	2	1.8
	Intermediate	2	1.8
	Susceptible	3	2.7
	Not tested	105	93.8

"*n*" represents the number of cases with a specific infection source among the total cases.

C/Sculture and susceptibility

The most common antibiotic used to treat ABB was a combination of colistin and meropenem in 56.3%, followed by meropenem alone in 18.8% (Table 7). Higher survival rates were observed with appropriate antibiotic therapy compared to inappropriate antibiotic therapy (45.6% vs 6.3%). The difference was found to be statistically significant (*P*<0.02) (Table 8).

**Table 7.** Antibiotics used, *n* (%)

Antibiotics	Frequency (*n*)	%
Cefepime	3	2.7
Ceftazidime	7	6.3
Ceftriaxone	1	0.9
Ciprofloxacin	4	3.6
Colistin and meropenem	63	56.3
Gentamicin and meropenem	6	5.4
Imipenem	1	0.9
Meropenem	21	18.8
Meropenem/colistin	1	0.9
Not mentioned	2	1.8
Piperacillin and tazobactam	3	2.7

"*n*" represents the number of cases with a specific infection source among the total cases.

**Table 8.** Appropriateness of antibiotics

Appropriateness	Total,*n* (%)	Alive, *n*	Dead, *n*	*P*-value*
Appropriate	57 (50.9)	26	31	<0.02
Inappropriate	16 (14.3)	1	15	
Unknown as antibiotic susceptibility was not tested or not mentioned	39 (34.8)	14	25	

The appropriateness of an antibiotic was determined based on the match between the susceptibility pattern of the bacteria in culture and the antibiotic treatment given.*Pearson’s Chi-square test.

*n*, number of patients.

* Pearson’s chi-square test.

*n*number of patients

While the study was being conducted, colistin was the recommended drug to treat CRAB. Interestingly, increased mortality was observed among those who had received empiric colistin (*n*=53/75, 70.7%) compared to other antimicrobials (*n*=18/37, 48.6%) (*P*=0.023) (Table 4).

## Discussion

A retrospective analysis of ABB was performed in a tertiary hospital in the Kingdom of Saudi Arabia, to become more aware of the prevalence, risk factors, clinical disease, antibiotic regimen, antibiotic susceptibility, treatment outcomes and mortality associated with the infection.

The study showed that most of the *Acinetobacter* isolates in this hospital were CRAB. The cause of infection is mostly CLABSI, followed by healthcare-acquired pneumonia. Co-infection with other MDR organisms, such as ESBL-producing Gram-negative organisms and CRE, was not uncommon. The overall mortality was 63.4% and was associated with various factors, such as hospital ward, higher age, duration of antibiotic treatment, inappropriate antimicrobial therapy and empirical use of colistin. We found that the mortality was considerably higher in patients with a high Charlson Comorbidity Index, Pitt bacteraemia score, or qSOFA score, which is not surprising as these are all known indicators of risk or disease severity.

Patients with CRAB had high mortality rates (74.4%). Other studies showed an overall mortality of patients with CRAB of 46–100% [40,45]. In a small study in Greece, 100% of the CRAB patients died (*n*=13/13) [40]; 30-day mortality was 64.5% in a study in Thailand [41] and 83.7% in Brazil [45]; mortality at discharge was 60.7% in Taiwan [43] and mortality at unspecified time points was 79.5% in China [42] and 46.4–55.8% in Turkey [44]. Therefore, the high mortality observed in our patients with CRAB was not unusual since previous studies have shown similar mortality rates. It may be because CRAB has limited treatment options and is often acquired in hospitals, where patients are already vulnerable as a result of other conditions.

The most common source of *Acinetobacter* bacteraemia in our patients was CLABSI. This is in line with other studies showing vascular catheters and the respiratory tract as the most common sources of *Acinetobacter* bacteraemia [46,48]. However, CLABSI was probably not associated with higher mortality because, in all patients in our study, the source control was applied by catheter removal, along with appropriate antibiotic use. Patients who had healthcare-acquired pneumonia as a source of bacteraemia were more likely to die; however, the difference was found to be insignificant. In a study of 401 patients with healthcare-acquired pneumonia (i.e. ventilation-associated pneumonia), CRAB showed that in these patients, the 21-day all-cause mortality rate was found to be 25.2% [49]. Other studies found that the mortality of healthcare-acquired ABB ranged between 20 and 40%, depending on comorbidities and disease severity as well as the appropriateness of initial therapy [40,48].

In our study, the resistance to meropenem was 68% lower than a study conducted in 2018–2019 in Saudi Arabia, which showed 94.6% meropenem resistance. This study, however, only analysed patients in ICUs, a selection that generally includes more severe patients, who may be more likely to have resistant bacteria [50]. The research focusing on ventilator-associated pneumonia in Saudi Arabia between 2005 and 2009 found a prevalence of 40.9% of MDR-*A. baumannii* [51]. In 2006, the prevalence of *A. baumannii* resistant to carbapenem was found to be 19% in a military hospital in Riyadh, Saudi Arabia [52] and 81.4% in 2006–2008 in another hospital [53].

We evaluated various factors that potentially affected mortality and found that it was much higher in patients who have a higher Pitt bacteraemia score, Charlson Comorbidity Index and qSOFA score. This was also observed in a study that looked at mortality for different *Acinetobacter* species and found similar results [54]. After 2020, a huge number of patients were found to be infected or colonized with organisms that were MDR. This increase in MDR can be attributed to the increased antibiotic usage at the time of the COVID-19 pandemic since many patients suffering from this infection were admitted to the ICU and received mechanical ventilation. These patients were given broad-spectrum antibiotics to prevent secondary bacterial infection, which can result in the emergence of MDR organisms. This phenomenon was also noted in a review of co-infections and secondary infections caused by *A. baumannii* in patients with COVID-19 [55].

Another important consideration is the antibiotic choice. We found that the most common antibiotic used was colistin–meropenem, which was also observed in another study [56]. Current guidelines recommend combination therapy to prevent resistance development for increased efficacy. The authors assessed a combination of colistin–tigecycline against a combination of colistin–carbapenem and found that patients receiving colistin–tigecycline combination showed a higher 14-day mortality rate [56]. We also assessed the appropriateness of the drug therapy and found that mortality was found to be significantly lower in patients receiving appropriate antibiotics. Studies in Brazil and Korea showed that the mortality rate also reduced in response to appropriate antimicrobial therapy [45,57]. Nevertheless, 43% of our patients died despite receiving appropriate therapy. This is not a new finding, as it was reported that the severity of infection measured by the Acute Physiology and Chronic Health Evaluation (APACHE) score, as well as host factors, are the primary determinants of the outcome rather than the appropriateness of antimicrobial therapy [58]. In another study, initiating appropriate therapy early in patients with ABB led to favourable outcomes [58].

Treatment with empirical colistin was associated (*P*<0.05) with high mortality (67%), which is consistent with several reports that colistin is associated with higher mortality due to toxicities observed with colistin [59,61]. To date, there is no standard treatment for CRAB, but the general consensus is to combine high-dose ampicillin–sulbactam along with another antimicrobial agent, such as minocycline, colistin, polymyxin B, cefiderocol or tigecycline [62]. Unfortunately, neither cefiderocol nor sulbactam (as a single agent) was widely available in Saudi Arabia when the study was conducted; hence, they were not used.

The present study is not exempted from limitations. First, the total number of patients included in the study was small, as ABB is not a common infection, which may have affected meaningful statistical analyses. Second, due to the single-centre nature of the study, the findings are not generalizable, and larger studies in multiple centres are needed for more robust data. Third, this was a retrospective study, which can have the potential for information bias. Finally, we have not performed genetic resistance testing of the CRAB isolates, as this technique is unavailable at our institution. Genetic testing can identify the genes responsible for the resistance pattern, which may help us understand the most common gene that confers resistance in the isolates at our institution.

The emergence of resistant *A. baumannii* strains has been increasing as evidenced by the elevating resistance rates over time. CRAB is a growing threat in hospitals in Saudi Arabia, especially in the critical care setting, and carries a very high risk of mortality. To prevent ABB-associated mortality, an appropriate regimen and duration of treatment are necessary. Hospitals must also practice proper hygiene to prevent the spread of ABB. Future studies must focus on novel ways to prevent CRAB infections and assess the use of new antimicrobials, such as cefiderocol or sulbactam–durlobactam, and other treatment options, such as bacteriophages, which provide promising new therapies in this field.

## Abbreviations

*A. baumannii*: *Acinetobacter baumannii*
ABB: *Acinetobacter baumannii* bacteraemia
BMI: body mass index
CLABSI: central line-associated bloodstream infection
CRAB: carbapenem-resistant *Acinetobacter baumannii*
CRE: carbapenem-resistant *Enterobacteriaceae*
ESBL: extended-spectrum β-lactamase
HAI: healthcare-acquired infections
ICU: intensive care units
MDR: multidrug resistant
MICU: medical intensive care units
qSOFA: quick sequential organ failure assessment
